# *De novo* Sequencing and Comparative Transcriptome Analyses Provide First Insights Into Polysaccharide Biosynthesis During Fruiting Body Development of *Lentinula edodes*

**DOI:** 10.3389/fmicb.2021.627099

**Published:** 2021-07-13

**Authors:** Qiaozhen Li, Jing Chen, Jianyu Liu, Hailong Yu, Lujun Zhang, Chunyan Song, Yu Li, Ning Jiang, Qi Tan, Xiaodong Shang, Yunfu Gu

**Affiliations:** ^1^Shanghai Key Laboratory of Agricultural Genetics and Breeding, Institute of Edible Fungi, Shanghai Academy of Agricultural Sciences, Shanghai, China; ^2^College of Resources, Sichuan Agricultural University, Chengdu, China

**Keywords:** *Lentinula edodes*, different development stages, comparative transcriptome, RNA-Seq, polysaccharide biosynthesis

## Abstract

Polysaccharides separated from *Lentinula edodes* are well known for their medicinal properties. However, the precise molecular mechanisms of polysaccharide biosynthesis in *L. edodes* remain unclear. In this study, the fruiting bodies of *L. edodes* in four developmental stages with significant differences in polysaccharide yield were collected, and the characteristics of polysaccharides were studied. *De novo* sequencing and comparative transcriptomic analysis were performed by using high-throughput Illumina RNA-sequencing. KS1P30, KS2P30, KS3P30, and KS4P30 were obtained from the four developmental stages, respectively, by hot water extraction and 30% ethanol precipitation. These four polysaccharides had good immune activity *in vitro*; all of them were β-glucopyranose with a high molecular weight. Glucose was the main monosaccharide component of these polysaccharides. High-quality clean reads (57.88, 53.17, 53.28, and 47.56 million for different growth stages) and mapping ratios ranging from 84.75 to 90.11% were obtained. In total, 11,493 (96.56%) unigenes and 18,924 (97.46%) transcripts were successfully annotated in five public databases. The biosynthetic pathway and related genes of LEFP30 were mined. The molecular mechanism of LEFP30 yield change in the different developmental stages was predicted. The results provide some insights into the possible mechanisms involved in the biosynthetic pathway of this kind of polysaccharide in *L. edodes* fruiting bodies. They also indicate that candidate genes can be used as important resources for biotechnology and molecular breeding to regulate *L. edodes* fruiting body polysaccharide biosynthesis.

## Introduction

*Lentinula edodes* (Berk.) Pegler, also called shiitake in Japan, is one of the most important edible and medicinal fungi in East Asia (Chen et al., 2015). It has good nutritional value and pharmacological activities (Bisen et al., 2010). Polysaccharides separated from *L. edodes* are well known for their medicinal properties, including antitumor, immunomodulating, antioxidant, anti-inflammatory, antimicrobial, and metabolic regulatory activities (Li W. et al., 2018; Ren et al., 2019). Different extraction and purification methods of polysaccharides lead to different molecular weights, primary structures, advanced structures, and pharmacological actions (Xu et al., 2014; Morales et al., 2019). At present, most of the polysaccharides isolated and purified from *L. edodes* are β-(1→3)-D-glucans as the main chain, with a different molecular weight (Chihara et al., 1970; Liu et al., 1998; Wang et al., 2014; Ren et al., 2018), and there is also a small amount of heteropolysaccharides (Carbonero et al., 2008; Wang et al., 2019). The branched (1→3)-β-D-glucans isolated from various fungi are thought to show immunomodulatory activity by activating the host immune system and/or increasing functional activity of macrophages (Bao et al., 2002; Carbonero et al., 2012; Liu et al., 2014). High molar mass, triple helix, and the β-(1→6)-branch are regarded as favorable structural parameters for their immunological activity (Cleary et al., 1999; Surenjav et al., 2006). The molecular weight and bioactivity of polysaccharides obtained by precipitation with a low alcohol concentration are higher than that of polysaccharides obtained by precipitation with a high alcohol concentration (Li Q. Z. et al., 2015; Wang J. M. et al., 2020).

The contents of crude polysaccharides are influenced by the cultivars and the characteristics of the environment (Kimmons et al., 2010). The extraction and purification technologies affect the yield of polysaccharides (Leong et al., 2020). Specifically, the yield of crude polysaccharide extract obtained from freshly harvested fruiting bodies varies from 260 to 825 mg/100 g in fresh weight (Monic et al., 2011). Owing to the low yield of *L. edodes* polysaccharides, the cost of large-scale production is high. Industrial production of polysaccharides is needed to increase the yield of polysaccharides and thereby reduce the cost. Genetic or metabolic engineering is a way to obtain a higher yield of polysaccharides. With the completion of the whole genome sequence of many mushrooms (Chen et al., 2012; Li H.Y. et al., 2018; Han M. et al., 2019; O’Connor et al., 2019), researchers have begun to reveal the biosynthesis regulation mechanism of polysaccharides in mushrooms at the molecular level (Ma et al., 2018). However, current research on improving polysaccharide production through genetic or metabolic engineering in edible fungi mainly focuses on *Ganoderma lucidum* (Tang and Zhong, 2002; Ji et al., 2015; Li M. et al., 2015; Xu et al., 2015; Li H.J. et al., 2016; Ma et al., 2018). Therefore, molecular breeding is expected to be an effective way to regulate polysaccharide accumulation in *L. edodes*. Moreover, understanding the pathway and the key enzyme genes of polysaccharide biosynthesis may aid in metabolic engineering to improve polysaccharide production.

Tang et al. (2013) used high-throughput Illumina RNA-sequencing to identify genes related to brown film formation by photoinduction in *L. edodes* (Tang et al., 2013). Cullen et al. (2016) completed and published the genome reference sequence and annotation information of *L. edodes* in 2016 (Cullen et al., 2016). Wang et al. (2018) used high-throughput Illumina RNA-sequencing to obtain the molecular mechanism of *L. edodes* fruiting body growth and development (Wang et al., 2018). More recently, researchers have used transcriptome approaches to discover and characterize genes involved in secondary metabolic pathways (Meena et al., 2016; Yu et al., 2016; Chen et al., 2018; Shan et al., 2020; Wang J. Q. et al., 2020).

Although certain enzymes are known to play roles in polysaccharide biosynthesis, the precise molecular mechanisms of the polysaccharide biosynthesis in *L. edodes* remain unclear. In previous studies (unpublished), we found that the yield of polysaccharides obtained from different development stages by hot water extraction and 30% ethanol precipitation was different. In the present study, comparative transcriptomic analysis of fruiting bodies from different development stages was performed by using high-throughput Illumina RNA-sequencing to predict the biosynthetic pathway and functional genes associated with polysaccharide LEFP30 in *L. edodes*. This study aimed to provide candidate key genes and pathways for improving the fruiting body polysaccharide of *L. edodes* and lay the foundation for the synthesis of related polysaccharides *in vitro*.

## Materials and Methods

### Materials

**FIGURE 1:**
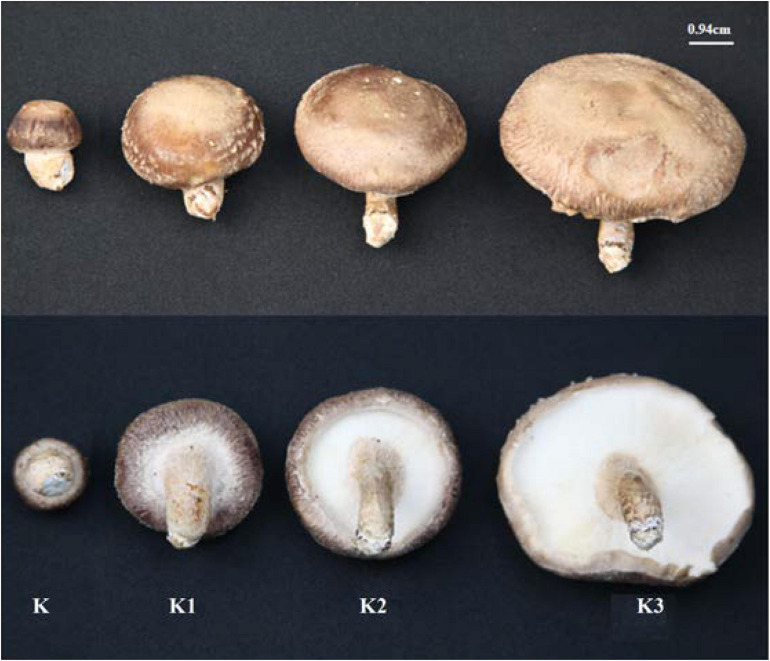
Morphological characteristics of *L. edodes* fruiting bodies at different development stages. K represented button stage, the fruiting body was undifferentiated, and pileus diameter was 0.5–1.0 cm. K1 represented harvest stage, the veil formed, and pileus diameter was 2.8–3.3 cm. K2 represented mature stage, and the veil dehisced completely. K3 represented opening stage, and the pileus opened completely.

**FIGURE 2:**
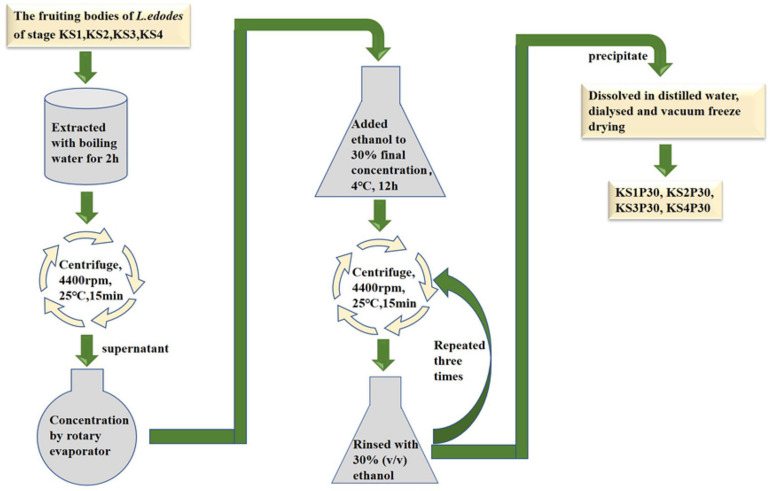
Preparation process of KS1P30, KS2P30, KS3P30, and KS4P30.

Strain F4 was provided by the Shanghai Edible Fungus Sub-center of the Agricultural Microorganism Preservation Center. The whole growth cycle of strain F4 is 90–95 days. The optimum temperature for mycelium growth is 21–23°C, and the temperature for fruiting is 17–18°C. Strain F4 was cultivated in polypropylene bags filled with a solid medium consisting of 79% oak sawdust, 20% wheat bran, and 1% plasters in Shanghai Guosen Biotechnology Co., Ltd. (Shanghai, China). Under aseptic conditions, two to three pieces of culture medium full of mycelium were picked with an inoculation shovel and inoculated into the cultivation bag. After inoculation with fungal mycelium, the bags were kept in the dark at 22–24°C and 65–70% relative humidity for 30 days, and then kept in the light at 22–24°C and 75–85% relative humidity for 60 days before being transferred to a ventilated field at 16°C and 90% relative humidity for 9 days. According to the previous experimental results, we selected fruiting bodies of four growth stages with significant differences in polysaccharide yield to harvest. According to the morphological characteristics shown in Figure 1, the fruiting bodies of the four stages were distinguished, and were named the button stage (K), harvest stage (K1), mature stage (K2), and opening stage (K3), respectively. After being dried in a laboratory drying oven at 55°C, the fruiting bodies were smashed and passed through a 0.18-mm sieve for the following experiment. At the same time, the fruiting bodies at different growth stages were cut off with a sterile scalpel and put into liquid nitrogen. KS1P30, KS2P30, KS3P30, and KS4P30 were prepared according to the schematic in Figure 2. Distilled water was added to the dried fruit body according to a material-to-liquid ratio of 1:18, extracted for 2 h by heating, and centrifugated for 15 min at 25°C and 4,400 rpm. The supernatant was concentrated by rotary evaporator and then we added ethanol to the concentrated liquid until the final concentration was 30% and placed it at 4°C for 12 h. It was then centrifugated for 15 min at 25°C and 4,400 rpm. The precipitate was washed three times by 30% ethanol and was finally dissolved in distilled water. Ethanol was removed by heating. Finally, it was dried by a freeze dryer.

### Polysaccharide Yield and Macrophage Activation

The yield of the polysaccharides was calculated by the ratio of the weight of the freeze-dried polysaccharide to the dry weight of fruit body. Macrophage activations of KS1P30, KS2P30, KS3P30, and KS4P30 were evaluated by measuring nitric oxide (NO) production using the Griess method (Li Q.Z. et al., 2016). Mouse macrophages (RAW264.7) were cultured in the DMEM medium containing 100 U/ml penicillin, 100 μg/ml streptomycin, and 10% fetal bovine serum at 37°C in a 5% CO_2_ humidified atmosphere. Aliquots (180 μl) of a RAW264.7 cell suspension (5 × 10^5^ cells/ml) were dispensed into each well of a 96-well plate together with 20 μl of the different test agents at 50, 250, and 500 μg/ml concentrations and incubated for 48 h. PBS and lipopolysaccharide (1 μg/ml) served as negative and positive controls, respectively. NO production was determined by measuring nitrite (NO_2_^–^) levels in cell supernatants using a colorimetric assay based on the Griess reaction. Supernatants (100 μl) were reacted with 50 μl of Griess reagent at room temperature for 10 min, and nitrite was determined by measuring the absorbance at 540 nm, using NaNO_2_ as the standard.

### High-Performance Size Exclusion Chromatography Analysis

All polysaccharides were dissolved in PBS (0.25% w/v) to analyze molecular weight by high-performance size exclusion chromatography (HPSEC). The system consisted of a 2414 refractive index detector (RI), a 2996 photodiode Array (UV) detector, a multiple-angle laser light-scattering detector (MALLS, DAWN HELEOS, Wyatt Technology, United States). The column was a TSK gel G6000 PW_*XL*_ (the separation range, 500,000–50,000,000) filtration column that was eluted with phosphate buffer (PB) (0.15 mol/L NaNO_3_ and 0.05 mol/L NaH_2_PO_4_, pH = 7) at a flow rate of 0.5 ml/min. The calibration of all detectors was done with Pullulan shodex standard (P-100, JM Science, Inc., NY, United States). Astra software (Version 6.1, Wyatt Technology Co., Ltd., United States) was utilized for data acquisition and analysis. The column temperature and RI detector temperature were maintained at 35°C.

### Monosaccharide Composition Analysis

Monosaccharide components and relative ratios in the four polysaccharides from different development stages were measured by high-performance anion-exchange chromatography (HPAEC) after hydrolyzing them with 2 M trifluoroacetic acid (TFA) for 3 h. The hydrolyzed polysaccharides were converted into alditol acetate derivatives, and the monosaccharides were analyzed by Dionex ICS2500 (Dionex, America) using a CarboPac^TM^ PA20 analytical column (3 mm × 150 mm). The composition and content of monosaccharides were detected by comparing the retention time and peak area of the tested residues with that of the monosaccharide standards (D-Glc, D-Gal, D-Ara, L-Fuc, L-Rha, D-Man, D-Xyl, and D-Fru) (Li Q.Z. et al., 2015).

### Infrared Spectra Analysis

The infrared spectra analysis of KS1P30, KS2P30, KS3P30, and KS4P30 was conducted based on the method described by Tie (Mei et al., 2008). After KS1P30, KS2P30, KS3P30, and KS4P30 were mixed with a KBr tablet, and the infrared spectrometer scan analysis was done with a resolution of 4 cm^–1^ and accumulated 32 times. The air background was deducted before scanning, and the scanning range was from 4,000 to 400 cm^–1^.

### RNA Sequencing

Three samples from each stage (button stage, harvest stage, mature stage, and opening stage) were chosen for RNA sequencing *via* Illumina Hiseq 4000 (Version 2 × 150 bp) at Shanghai Majorbio Bio-pharm Biotechnology Co., Ltd. (Shanghai, China). There were four stages, which were named stage K (button stage) (K_1, K_2, and K_3), stage K1 (harvest stage) (K11, K12, and K13), stage K2 (mature stage) (K21, K22, and K23), and stage K3 (opening stage) (K31, K32, and K33).

#### Total RNA Extraction, cDNA Library Preparation, and Illumina Sequencing

Total RNA was isolated from 12 fruiting body pileus samples using the TRIzol^®^ Reagent (Invitrogen, Carlsbad, CA, United States) according to the manufacturer’s instructions (TaKara). Then, the quality and quantity of RNA were determined using a 2100 Bioanalyzer (Agilent Technologies, Santa Clara, CA, United States) and a ND-2000 (Thermo Fisher Scientific, Waltham, MA, United States). Only the high-quality RNA sample (OD260/280 = 1.8–2.2, OD260/230 ≥ 2.0, RIN ≥ 6.5, 28S:18S ≥ 1.0, > 1 μg) was used to construct the sequencing library. The RNA-seq transcriptome library was prepared following the TruSeq^TM^ RNA sample preparation kit from Illumina (San Diego, CA, United States) using 1 μg of total RNA. Shortly afterward, the messenger RNA was isolated according to polyA selection method by oligo (dT) beads and then fragmented by the fragmentation buffer first. Second, double-stranded cDNA was synthesized using a SuperScript double-stranded cDNA synthesis kit (Invitrogen, CA, United States) with random hexamer primers (Illumina). Then, the synthesized cDNA was subjected to end repair, phosphorylation, and “A” base addition according to Illumina’s library construction protocol. Libraries were size-selected for cDNA target fragments of 300 bp on 2% Low Range Ultra Agarose and then PCR-amplified using Phusion DNA polymerase (NEB) for 15 PCR cycles. After being quantified by TBS380, the paired-end RNA-seq sequencing library was sequenced with the Illumina HiSeq xten/NovaSeq 6000 sequencer (2 × 150 bp read length).

#### Transcriptome Sequencing Data Processing

SeqPrep^1^ and Sickle^2^ were used to remove reads with adapter sequences, low quality, ambiguous bases, ‘‘N,’’ and less than 30 bp. Hisat2^3^ was used to align clean reads to *L. edodes* genome (*L. edodes*-v1.0^4^). The mapped reads were assembled with StringTie^5^, and blastx alignment (e-value < 0.00001) was performed between all the unigenes and transcripts obtained by transcriptome assembly with five databases (GO, KEGG, NR, Pfam, and EggNOG) to acquire the functional information of the unigenes and transcripts comprehensively and obtain statistics on the annotation of each database (Liu and Zhu, 2020).

#### Differentially Expressed Gene (DEG) Analysis

Transcripts per million reads (TPM) scores for the transcripts were calculated using RSEM^6^. DEGs were identified through pairwise comparisons of the four stages by DESeq2^7^, including group KvsK1, KvsK2, KvsK3, K1vsK2, K1vsK3, and K2vsK3. The statistical significance of gene expression differences was evaluated using an adjusted *p* < 0.05 and a |log2FC| ≥ 1 as the threshold.

#### GO and KEGG Pathway Enrichment Analysis

Gene Ontology (GO) and Kyoto Encyclopedia of Genes and Genomes (KEGG) pathway enrichment analyses of DEGs were performed by Goatools software using the Fisher exact test and RStudio, respectively. The calculation principle was the same. GO terms and KEGG pathways with adjusted *p* < 0.05 were considered significantly enriched.

### Quantitative RT-PCR (qRT-PCR) Validation

#### RNA Extraction and cDNA Library Construction

RNA was extracted by the MiniBEST Plant RNA Extraction Kit (TaKaRa, China) based on the manufacturer’s instructions. Fruiting bodies of *L. edodes* at the button stage, harvest stage, mature stage, and opening stage were used as materials. Then, cDNA was synthesized using total RNA as the template by gDNA Removal and the cDNA Synthesis SuperMix Kit (TransGen, China).

#### qRT-PCR Validation

The quality and quantity of RNA were determined by the same method as above. The integrity of the RNA samples was measured by agarose gel electrophoresis. If the 28S and 18S bands could be observed clearly, the RNA samples had high integrity.

Quantitative RT-PCR analysis was carried out using total RNAs from the fruiting bodies of *L. edodes* at four development stages. Reverse transcription and quantification of 2 μg total RNA were carried out by a TransStart Top Green qPCR SuperMix Kit (TransGen, China) and 18S rRNA endogenous control. Gene-specific primers were designed using Primer Premier 6 software (Premier Biosoft International, Palo Alto, CA). Primer information was given in Supplementary Table 1. The PCR conditions were as follows: 94°C for 30 s, 94°C for 5 s, 60°C for 20 s, and 72°C for 10 s (40 cycles). The gene expression levels were determined using quantitative real-time PCR assays in 96-well plates using the ABI QuantStudio 6 Flex system (Applied Biosystems). The 2^–ΔΔ*Ct*^ method was used to calculate and normalize the relative expression of each gene (Livak and Schmittgen, 2001). Three technique replicates were performed using three biological samples.

### Statistical Analysis

All experiments were performed in triplicate. The biochemical analysis was performed in three replicate samples for each treatment, and the RNA-Seq assay was performed in triplicate for each treatment. Differences were considered statistically significant when *p* < 0.05. Statistical analysis was performed using IBMSPSS 25.0 (SPSS, Inc., Chicago, IL, United States).

## Results

### Isolation, Yields, and Macrophage Activation of Polysaccharides Obtained From Different Development Stages

**FIGURE 3:**
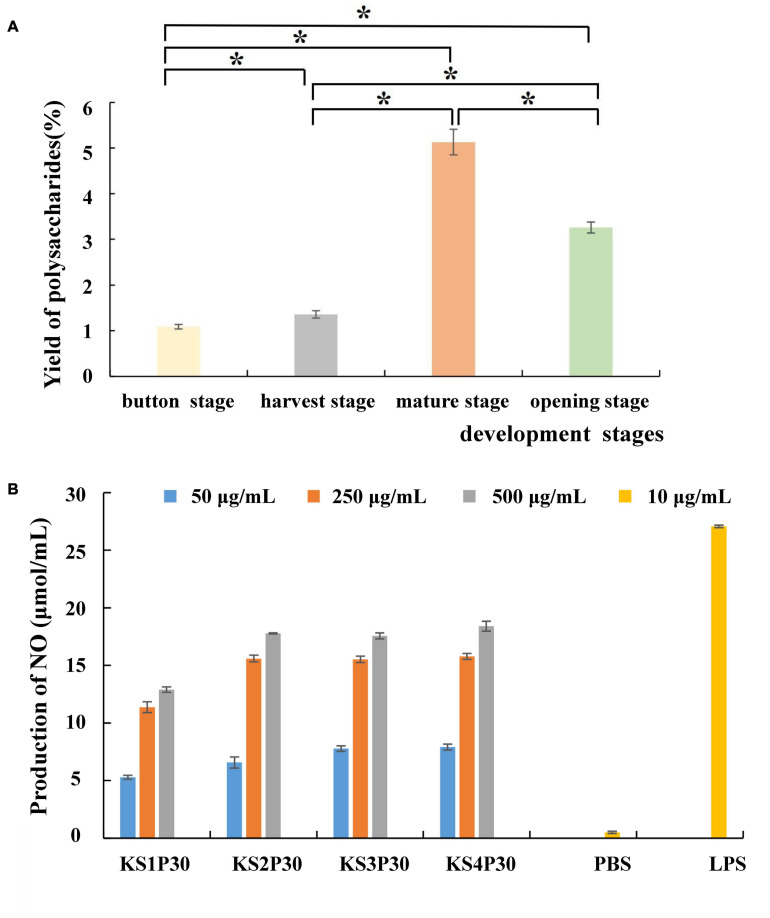
Yield and macrophage activation of LEFP30 obtained from different development stages. **(A)** Yields of polysaccharides. *Means that polysaccharide yield between the two was significantly different (*p* < 0.05). **(B)** Effect of polysaccharides obtained from different development stages on nitric oxide (NO) synthesis in murine macrophage-like cells RAW264.7 cells (5 × 10^5^ cells/ml).

Most polysaccharides of *L. edodes* were purified by columns such as DEAE cellulose chromatography and Sephadex series size-exclusion chromatography (Zhang et al., 2010; Qian et al., 2018; Wang et al., 2019). In this study, KS1P30, KS2P30, KS3P30, and KS4P30 were obtained in the button stage, harvest stage, mature stage, and opening stage, respectively, by low-concentration ethanol precipitation of hot water extract (Figure 2). The polysaccharide yields of *L. edodes* fruiting bodies at different development stages are shown in Figure 3A. There were significant differences in the yields of polysaccharides obtained from the different development stages. The yield of polysaccharides first increased in the button stage, harvest stage, and mature stage and were high-yield in the mature stage. Then, they decreased.

The antitumor effects of mushroom-derived polysaccharides involve the activation of the complementary system and macrophage-dependent systems (Lee et al., 2008). The immune activity of activated macrophages can be judged by the content of NO released by macrophages (Mackay and Pussell, 1986). All the polysaccharides examined in this study stimulated NO production by RAW264.7 macrophages (Figure 3B). The four polysaccharides showed good NO-stimulating activity. However, KS1P30 exhibited lower NO-stimulating activity than the other three polysaccharides at all three concentrations.

### Characteristics of Polysaccharides Obtained From Different Development Stages

**FIGURE 4:**
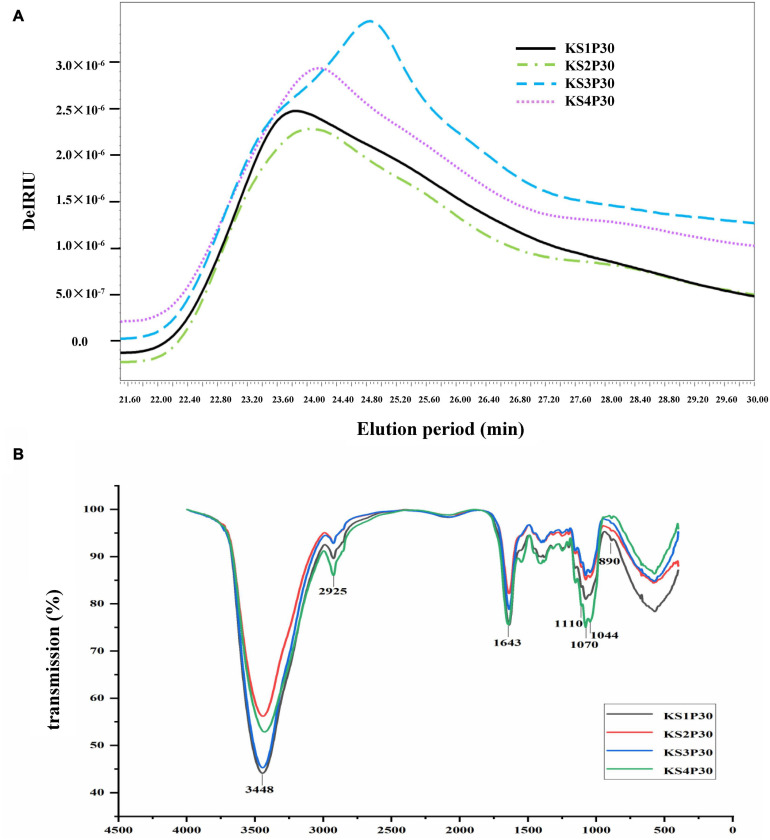
Characteristics of polysaccharides obtained from different development stages. **(A)** Molecular weight distribution of polysaccharides. KS1P30, KS2P30, KS3P30, and KS4P30 represented 30% alcohol-precipitation polysaccharides obtained from the button stage, harvest stage, mature stage, and opening stage respectively. **(B)** IR spectrum of KS1P30, KS2P30, KS3P30, and KS4P30.

The HPSEC profiles (Figure 4A) indicate that KS1P30, KS2P30, KS3P30, and KS4P30 were a single peak. HPSECMALLS-RI was used to analyze its molecular weight. The weights of the polysaccharides were calculated by normalizing the laser at different angles. The average molecular weight (M_*W*_) of KS1P30, KS2P30, KS3P30, and KS4P30 was 4.511 × 10^6^, 4.099 × 10^6^, 1.027 × 10^7^, and 7.718 × 10^6^, respectively (Table 1). The polydispersity index could measure the width of molecular weight distribution (Rogosic et al., 1996). In this study, the polydispersity index (M_*W*_/M_*n*_) of KS3P30 was closest to 1 (Table 1), which belonged to the narrow distribution sample, indicating that the KS3P30 had a relatively higher purity.

**TABLE 1:** Molecular weight distribution of 30% ethanol precipitated polysaccharides obtained from *L. edodes* at different development stages.

30% ethanol polysaccharides	Weight-average molecular weight Mw (Da)	Number-average molecular weight Mn (Da)	Polydispersity index Mw/Mn
KS1P30	4.511 × 10^6^	2.537 × 10^6^	1.778
KS2P30	4.099 × 10^6^	2.922 × 10^6^	1.403
KS3P30	1.027 × 10^7^	8.636 × 10^6^	1.189
KS4P30	7.718 × 10^6^	2.525 × 10^6^	3.056

*KS1P30, KS2P30, KS3P30, and KS4P30 represented 30% alcohol-precipitation polysaccharides obtained from the button stage, harvest stage, mature stage, and opening stage, respectively.*

The results of high-performance anion exchange chromatography (HPAEC) showed that the monosaccharide in KS1P30, KS2P30, KS3P30, and KS4P30 consisted of glucose, fucose, galactose, and mannose. The monosaccharide (Table 2) indicated that the mole ratio of fucose, galactose, glucose, and mannose in KS1P30, KS2P30, KS3P30, and KS4P30 was 1:11:63:5, 1:9:63:4, 1:9:60:4, and 1:7:63:5, respectively.

**TABLE 2:** Monosaccharide composition of 30% ethanol precipitated polysaccharides obtained from *L. edodes* at different development stages.

30% ethanol polysaccharides (mol%)	Fucose	Galactose	Glucose	Mannose
KS1P30	1.25	13.75	78.75	6.25
KS2P30	1.30	11.69	81.82	5.19
KS3P30	1.35	12.16	81.08	5.41
KS4P30	1.32	9.21	82.89	6.58

*KS1P30, KS2P30, KS3P30, and KS4P30 represented 30% alcohol-precipitation polysaccharides obtained from the button stage, harvest stage, mature stage, and opening stage, respectively.*

Fourier transform-infrared (FT-IR) spectroscopy was used to determine functional groups and configuration of KS1P30, KS2P30, KS3P30, and KS4P30. The results showed that the four polysaccharides had similar absorption spectra (Figure 4B). The spectra showed absorption at 3,448, 2,925, 1,643, 1,070, and 890 cm^–1^, corresponding to the stretching of the O-H, C-H, C = C stretch, or C = O pyranoside bond groups and β-glycoside bonds (Kacurakova et al., 2000). These results demonstrated that all four polysaccharides belonged to β- pyranoside polysaccharides.

The identification results of polysaccharides suggest that the four polysaccharides are β-glucans with fucose, galactose, and mannose branches. This kind of polysaccharide is named LEFP30.

### RNA Sequencing and Transcriptomic Assembly

The systematic analysis of gene expressions in *L. edodes* transcriptome at different development stages was performed by high-throughput sequencing. Approximately 58.28, 54.00, 54.14, and 48.35 million raw reads were obtained from stages K, K1, K2, and K3 separately (Supplementary Table 2). After quality control of raw data, 98.37–99.31% of high-quality clean reads (57.88, 53.17, 53.28, and 47.56 million for different development stages) were reserved for mapping to the *L. edodes* reference genome sequence (*L. edodes*-v1.0) and subsequent analysis. The detailed mapping consequence was demonstrated in Supplementary Table 2. Mapping ratios of all test groups ranged from 84.75 to 90.11%, indicating that a high level of gene expression was found in each group.

### Gene Annotation and Functional Classification

**FIGURE 5:**
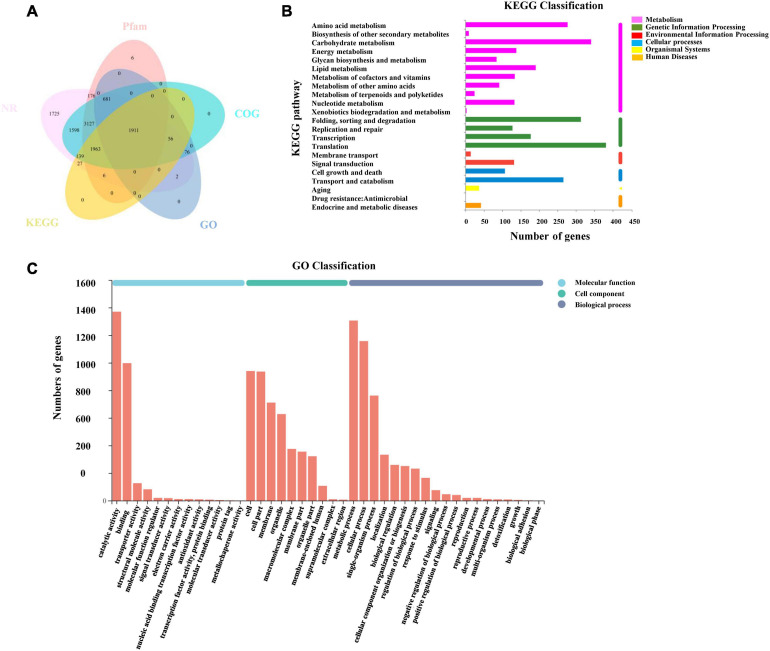
Annotation information of assembled unigenes in *L. edodes*. **(A)** Venn diagram of the distribution of annotation information from five public databases. **(B)** KEGG classification of unigenes in *L. edodes*. **(C)** GO classification of unigenes in *L. edodes*.

All transcripts and unigenes acquired from the transcriptome assembly were compared with five databases (GO, KEGG, NR, Pfam, and COG) to gain comprehensive annotation information on transcripts and genes (Figure 5A). Moreover, 18,924 (97.46%) transcripts and 11,493 (96.56%) unigenes were successfully annotated (Supplementary Table 3).

Blast2go was used to obtain the unigene function terms through the GO database. Among 11,902 unigenes, we annotated 2,726 (22.9%) unigenes. These genes were divided into three GO categories and 42 sub-categories (Figure 5C). The metabolic process (1,306, 10.97%), cellular process (1,157, 9.72%), single-organism process (762, 6.4%), localization (333, 2.8%), biological regulation (259, 2.18%), cellular component organization or biogenesis (251, 2.11%), and other sub-categories (636, 5.34%) were included in biological process categories. Cellular component categories contained the cell (940, 7.9%), cell part (936, 7.86%), membrane (711, 5.97%), organelle (628, 5.28%), and other sub-categories (1,176, 9.88%). Molecular function categories contained binding (997, 8.38%), catalytic activity (1,370, 11.51%), transporter activity (126, 1.06%), and other sub-categories (165, 1.39%) (Supplementary Table 4).

Then, 4,102 unigenes were annotated and classified into six KEGG categories and 22 pathways (Figure 5B). KEGG pathways contained translation (367, 3.08%); carbohydrate metabolism (336, 2.82%); folding, sorting, and degradation (300, 2.52%); amino acid metabolism (272, 2.29%); transport and catabolism (253, 2.13%); and other pathways (Supplementary Table 5).

### Identification of DEGs and Enrichment Analysis of Transcripts

**FIGURE 6:**
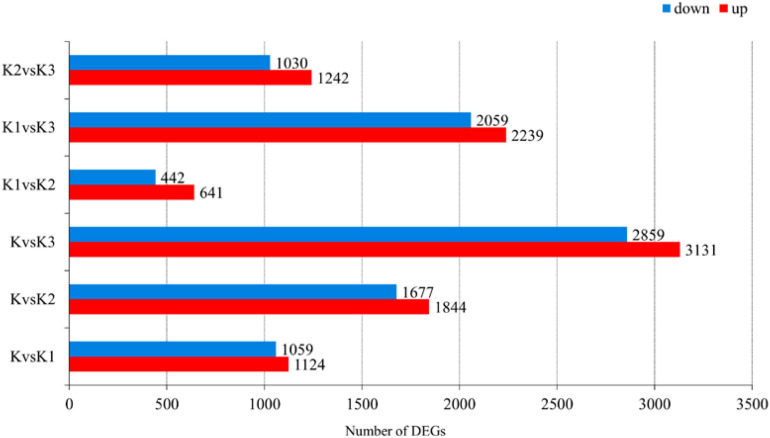
Number of differentially expressed genes (DEGs) in *L. edodes* fruiting bodies at different development stages. The *y*-axis represents the comparison sample name. K, K1, K2, and K3 represented the button stage, harvest stage, mature stage, and opening stage, respectively.

Pairwise comparisons at adjusted *p* ≤ 0.05 and log2Fold Change ≥ 1 or ≤ −1 were made to confirm the amount of DEGs in the four development stages. Then, 7,325 genes were differentially expressed, and the number of downregulated DEGs was lower than the number of upregulated DEGs during development (Figure 6 and Supplementary Table 6). The functionality of those genes included information storage and processing, cellular process and signaling, and metabolism.

**FIGURE 7:**
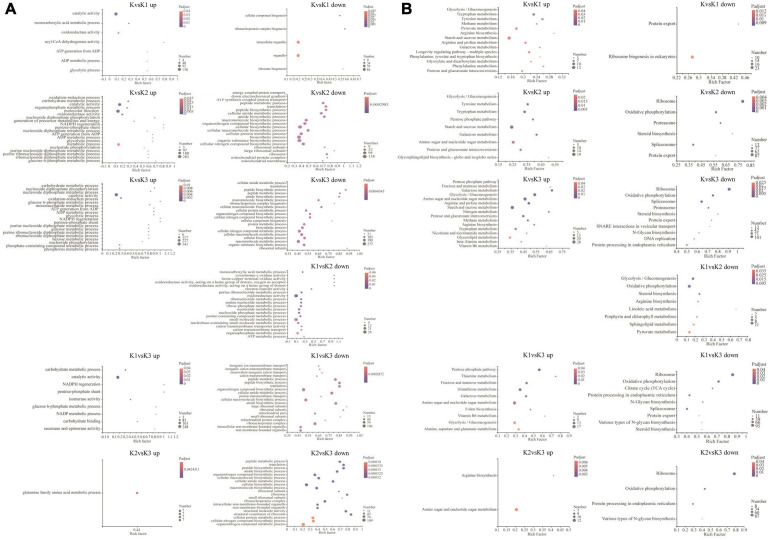
GO enrichment analysis **(A)** and KEGG enrichment pathway analysis **(B)** of the differentially expressed genes (DEGs). GO and KEGG enrichment were filtered using the significant criterion of corrected *p* < 0.05.

Gene ontology enrichment was filtered using the significant criterion of corrected *p* < 0.05. The yield of polysaccharide increased significantly in K2, but decreased significantly in K3. Therefore, in the comparative analysis of differential genes, we mainly analyzed the genes that were significantly upregulated in K2 compared to K and were significantly downregulated in K3 compared to K2. The GO entries showed that the significantly upregulated genes in K2 compared to K were enriched in 20 terms, such as the carbohydrate metabolic process, oxidoreductase activity, catalytic activity, ATP generation from ADP, the ADP metabolic process, the glucose 6-phosphate metabolic process, and the pentose-phosphate shunt. The significantly downregulated genes in K3 compared to K2 were enriched in 20 terms – for example, the cellular macromolecule biosynthetic process, cellular biosynthetic process, and ribosomes (Figure 7A and Supplementary Table 7).

Kyoto encyclopedia of genes and genomes pathway enrichment analysis of DEGs was carried out to further comprehend the biological processes arising during the development of *L. edodes* fruiting bodies (Figure 7B and Supplementary Table 8). KEGG pathway enrichment showed that the significantly upregulated genes in K2 compared to K were enriched in nine terms (i.e., glycolysis/gluconeogenesis, pentose phosphate pathway, starch and sucrose metabolism, galactose metabolism, amino sugar and nucleotide sugar metabolism, pentose and glucuronate interconversions, and glycosphingolipid biosynthesis). The significantly downregulated genes in K3 compared to K2 were enriched in ribosomes, oxidative phosphorylation, protein processing in the endoplasmic reticulum, and various types of N-glycan biosynthesis.

### Expression of Genes Involved in Hypothesized Mushroom Nucleotide Sugar Precursors Biosynthetic Pathways

**FIGURE 8:**
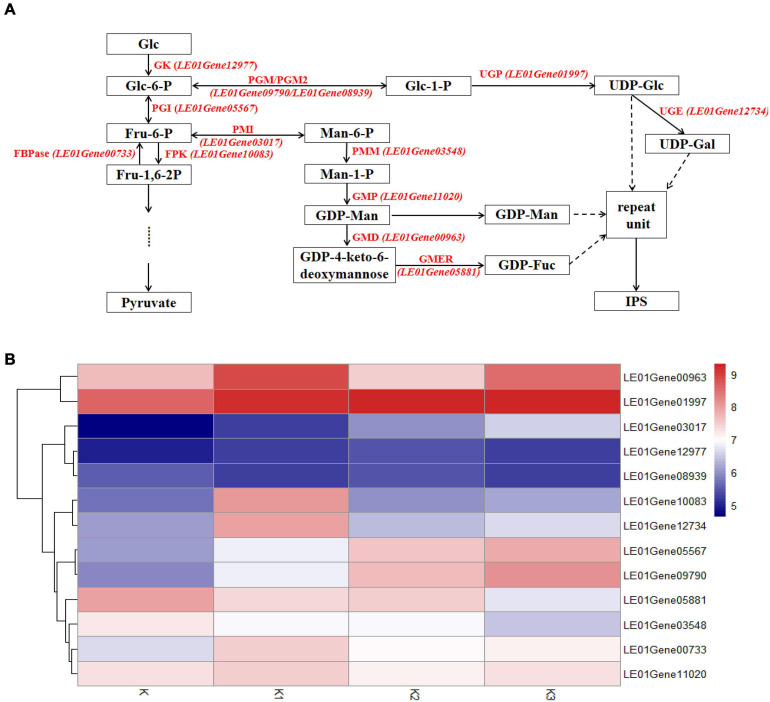
Expression of related genes and hypothesized nucleotide sugar precursors biosynthetic pathways in *L. edodes*. **(A)** The mushroom nucleotide sugar precursor biosynthetic pathways modified from reference. GK, Glucokinase; PGI, Phosphoglucose isomerase; FBPase, Fructose-1,6-biphosphatase; FPK, Phosphofructokinase; PMI, Phosphomannose isomerase; PMM, Phosphomannose mutase; GMP, GDP-Man pyrophosphorylase; GMD, GDP-Man dehydratase; GMER, GDP-4-keto-6-deoxymannose epimerase/reductase; PGM/PGM2, Phosphoglucose mutase; UGP, UDP-Glc pyrophosphorylase; UGE, UDP-Gal-4-epimerase. **(B)** Heatmap analysis of expressed mRNA involved in mushroom nucleotide sugar precursor biosynthetic pathways.

The transcriptomes were analyzed to identify genes involved in mushroom polysaccharide metabolism. Identification was performed using the hypothesized mushroom nucleotide sugar precursors biosynthetic pathways according to Wang et al. (2017b). Thirteen homologous genes were obtained (Figure 8A). Heatmap analysis of the mRNA genes involved in polysaccharide biosynthetic pathways is shown in Figure 8B.

#### Glycolysis

Genes encoding glucokinase (GK), phosphoglucose isomerase (PGI), fructose-1,6-biphosphatase (FBPase), and phosphofructokinase (FPK) from glycolysis were observed. The gene encoding PGI (*LE01Gene05567*) was upregulated during the developmental stages. The other three genes were upregulated in K1 compared to K, and the gene encoding GK (*LE01Gene12977*) was downregulated in stage K3; however, the genes (*LE01Gene00733 and LE01Gene10083*) encoding FBPase and FPK were downregulated in K2.

#### GDP-Man and GDP-Fuc Synthetic Pathways

Gene expressions encoding phosphomannose isomerase (PMI), phosphomannose mutase (PMM), GDP-Man pyrophosphorylase (GMP), GDP-Man dehydratase (GMD), GDP-4-keto-6-deoxymannose epimerase/reductase (GMER) from GDP-Man, and GDP-Fuc synthetic pathways are shown in Figure 8B. The gene encoding PMI (*LE01Gene03017*) was upregulated during the developmental stages. In contrast, the genes encoding PMM and GMER (*LE01Gene03548 and LE01Gene05881*) were downregulated during the developmental stages. The gene encoding GMP (*LE01Gene11020*) was upregulated in K1 compared to K and K3 compared to K2, and was downregulated in K2 compared to K1.

#### UDP-Glc and UDP-Gal Synthetic Pathways

Gene expressions encoding phosphoglucose mutase (PGM), phosphoglucose mutase 2 (PGM2), UDP-Glc pyrophosphorylase (UGP), and UDP-Gal-4-epimerase (UGE) from UDP-Glc and UDP-Gal synthetic pathways were observed. Genes encoding PGM and PGM2 (*LE01Gene09790 and LE01Gene08939*) were upregulated during the developmental stages. The genes encoding UGP and UGE (*LE01Gene01997 and LE01Gene12734*) were upregulated in K2 compared to K1, and were downregulated in K3 compared to K2.

### Expression of Genes Involved in *β*-Glucan Synthesis in Fungi

**FIGURE 9:**
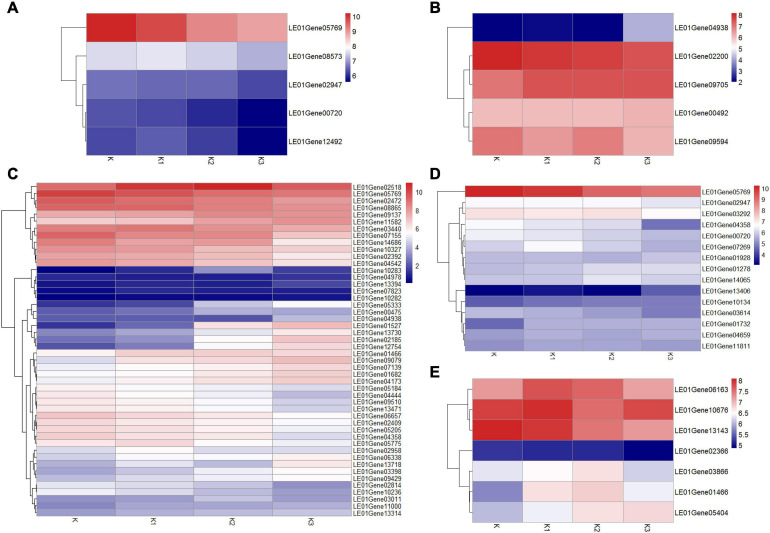
Heatmap analysis of expressed mRNA genes involved in the β-glucan synthesis. **(A)** Expression of genes involved in β-1,3-glucan synthesis key enzymes. **(B)** Expression of genes involved in β-1,6-glucan synthesis key enzymes. **(C)** Expression of DGEs. **(D)** Expression of genes involved in cell wall stress of MAPK signaling pathway-yeast. **(E)** Expression of genes involved in glycogen accumulation of longevity regulating pathway-yeast.

Considering that about 80% of the glucose ratio in the monosaccharide composition and β-configuration glycosidic bonds in the four polysaccharide fractions were obtained from the development stages K, K1, K2, and K3 (Table 2 and Figure 4B), we conducted an analysis of the β-glucan biosynthetic process in fungi to explore the regulatory pathway of glucan production. Therefore, 143 homologous genes in *L. edodes* were obtained using Blastp (1e−5) of the sequences in the fungi β-glucan biosynthetic process (GO: 0051274) (Supplementary Table 9). Heatmap analysis of the expressed mRNA genes involved in the β-glucan synthesis is shown in Figure 9.

#### Expression of Genes Involved in β-1,3-Glucan Synthesis Key Enzymes

Five expressed unigenes (*LE01Gene00720, LE01Gene02947, LE01Gene05769, LE01Gene12492*, and *LE01Gene08573*) encoding β-1,3-glucan synthesis key enzymes (β-1,3-glucan synthase, glucan synthesis regulator, and β-1,3-glucanosyltransferase) were identified in *L. edodes*. Moreover, the β-1,3-glucan synthase regulatory subunit gene (*LEGene 0105769*) was significantly downregulated in K2 and K3 compared to K1. The other β-1,3-glucan synthesis enzyme genes were not differentially expressed during the development of *L. edodes* fruiting bodies (Figure 9A).

#### Expression of Genes Involved in β-1,6-Glucan Synthesis Key Enzymes

Five homologous genes (*LE01Gene04938, LE01Gene02200, LE01Gene00492, LE01Gene09594*, and *LE01Gene09705*) of the β-1,6-glucan synthesis key genes SKN1 and KRE6 in fungi were identified in *L. edodes*. β-1,6-glucan synthesis-associated protein (SKN1) gene (*LE01Gene04938*) was significantly upregulated in stage K3 compared to other stages. The other genes were not differentially expressed during the development of *L. edodes* fruiting bodies (Figure 9B).

#### Expression of Differential Genes in Different Developmental Stages

The differentially expressed homologous genes involved in β-glucan synthesis in fungi at different developmental stages are shown in Supplementary Table 10. Heatmap analysis of the expressed mRNA genes involved in DEGs is shown in Figure 9C. Eight significantly upregulated genes were found in K1 compared to K, which were annotated as protein kinase, heat shock protein HSS1, negative regulator of sexual conjugation and meiosis, the dynein heavy chain, β-glucan synthesis-associated protein, probable α/β glucosidase agdC, serine/threonine protein kinase HSL1, and protein serine threonine kinase. Five DEGs were found in K1vsK2, and four genes were significantly upregulated, which were annotated as CBL-interacting protein kinase 6, serine/threonine protein kinase HSL1, the negative regulator of sexual conjugation and meiosis, and protein serine threonine kinase. Seven DEGs were found in K2vsK3, and four were significantly downregulated, which were annotated as heat shock protein HSS1, heat shock protein 70, heat shock protein 70, and Rho GTPase-activating protein 22. There were 41 DEGs in KvsK2, 22 of which were significantly upregulated. Then, 22 upregulated genes were enriched in two metabolic pathways: galactose metabolism and the longevity-regulating pathway. The response regulator receiver protein RIM15 gene (*LE01Gene01466*) and heat shock 70-kDa protein (*LE01Gene02518*) were enriched in the longevity-regulating pathway.

### Expression of Genes Involved in Cell Wall Stress of the MAPK Signaling Pathway of Yeast

Fifteen homologous genes involved in cell wall stress of the MAPK signaling pathway of yeast were identified in *L. edodes* (Figure 9D). GTP-binding protein rhoA gene (*LE01Gene05769*) was significantly downregulated in K2 compared to K and in K3 compared to K. The cell wall integrity and stress response component WSC gene (*LE01Gene13406*) were significantly upregulated in K3 compared to K, K1, and K2. Moreover, the Rho GTPase-activating protein gene (*LE01Gene04358*) was significantly downregulated in K3 compared to K, K1, and K2. There was no significant difference in the expression of other genes involved in the cell wall stress of the MAPK signaling pathway of yeast.

### Expression of Genes Involved in the Glycogen Accumulation of the Longevity-Regulating Pathway of Yeast

Seven homologous genes involved in the glycogen accumulation of the longevity-regulating pathway of yeast were identified in *L. edodes* (Figure 9E). The response regulator receiver protein RIM15 gene (*LE01Gene01466*) was significantly upregulated in K2 compared to K. There was no significant difference in the expression of other genes involved in glycogen accumulation of the longevity-regulating pathway of yeast.

### qRT-PCR Validation

**FIGURE 10:**
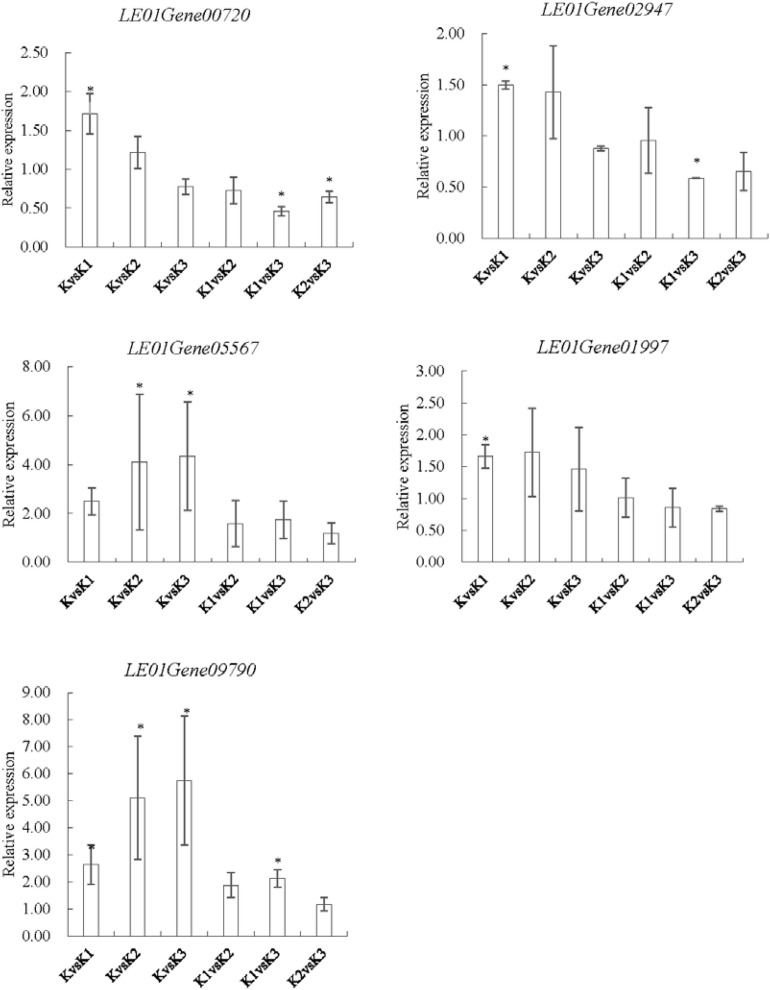
qRT-PCR analysis of five genes encoding enzymes related to the LEFP30 biosynthesis in *L. edodes*. *LE01Gene00720* and *LE01Gene02947* encoding 1,3-β-glucan synthase, *LE01Gene05567* encoding glucose-6-phosphate isomerase, *LE01Gene01997* encoding UDP-glucose pyrophosphorylase, and *LE01Gene09790* encoding phosphoglucomutase.^∗^*p* < 0.05, the expression of the unigene was significantly upregulated or downregulated in the treatment group, respectively.

To validate the expression data obtained by RNA sequencing, five genes were randomly selected for qRT-PCR validation (Figure 10). Their relative expression was normalized by 18S (internal reference gene, sequence, F-GCCTGGAA GTTTTGACG; R-TCCGAAGAGCAGAATGAA) expression (Xiang et al., 2018). Results showed that the expression pattern profiling of these unigenes by qRT-PCR was generally in accordance with those gained by RNA-Seq, suggesting that reliable expression results were generated *via* RNA-seq.

## Discussion

### LEFP30 Characteristics

Polysaccharides separated from *L. edodes* are well known for their medicinal properties. It was reported that the conformation (Wang et al., 2017a), M_*W*_ (Sletmoen and Stokke, 2008), and solubility of polysaccharides could significantly influence antitumor and immunomodulatory activities. The molecular weight and bioactivity of polysaccharides obtained by precipitation with a low alcohol concentration were higher than that of polysaccharides obtained by precipitation with high alcohol concentration (Li Q. Z. et al., 2015; Wang J. M. et al., 2020), which might be related to the content, physicochemical properties, and structure of polysaccharides (Maeda et al., 1988; Wang et al., 2014; Wang J. Q. et al., 2020; Zhao et al., 2018). Four polysaccharides, KS1P30, KS2P30, KS3P30, and KS4P30, were obtained by water extraction, precipitation with 30% alcohol, and repeated alcohol washing. Moreover, there were significant differences in the yields of polysaccharides. The polysaccharide contents of all four polysaccharides were more than 80%. These four polysaccharides were all β-glucopyranose with a high molecular weight. Glucose was the main monosaccharide component of these polysaccharides, but they also contained a small amount of fucose, galactose, and mannose. These results suggested that the four polysaccharides are probably β-glucans with fucose, galactose, and mannose branches. This kind of polysaccharide is named LEFP30.

At present, the polysaccharides isolated from the fruiting bodies of *L. edodes* are not only dextran, but also heteropolysaccharides (Xu et al., 2014). Shida et al. (1975) isolated a heterogalactan with α-(1→6)-D-galactopyranose as the main chain, parts of which are substituted in 2-position with fucose or mannose. Carbonero et al. isolated a heterogalactan with (1→6)-linked-α-D-galactopyranosyl units as the main chain, and it is partially substituted at O-2 by single-unit D-manp or L-fucp side chains; M_*W*_ is 16.2 × 10^3^ (Carbonero et al., 2008). Zheng and Geng (1995) isolated a polysaccharide, which is β-(1→3)-linked backbone with (1→6)-linked side chain and xylan, mannose, and galactose side chain, Xyl:Man:Glc:Gal = 5:6:75:5. At present, we have not found that the LEFP30 may be the same as the polysaccharides isolated from the fruiting bodies of *L. edodes* that has been identified. So, detailed structural characteristics need more research and further exploration in the future.

### LEFP30 Biosynthesis

The polysaccharide yield of *L. edodes* at different development stages was significantly different. The polysaccharide yield increased significantly in the harvest stage (K2) and then decreased significantly in the opening stage (K3). This suggested that the mechanism of polysaccharide synthesis or degradation may be different throughout development. Therefore, *L. edodes* fruiting bodies at different development stages were subjected to RNA-Seq analysis. By analyzing the transcriptomes, we found candidate genes and gained some insights into possible mechanisms involved in the biosynthetic pathway. High-quality clean reads (57.88, 53.17, 53.28, and 47.56 million for different growth stages) and mapping ratios ranging from 84.75 to 90.11% were obtained. In total, 11,493 (96.56%) unigenes and 18,924 (97.46%) transcripts were successfully annotated in five public databases. The GO results indicate that the DEGs that participate in many biological processes, as well as the carbohydrate metabolic process, oxidoreductase, and catalytic activity, may be closely related to the synthesis of polysaccharides, which agrees with previous reports (Cheng et al., 2016). According to the KEGG results, the DEG participation in many biological pathways, including many carbohydrate metabolism, glycan biosynthesis and metabolism, and the longevity-regulating pathway of multiple species, is also worthy of attention, and may be closely related to the synthesis of polysaccharides according to previous reports (Swinnen et al., 2006).

The biosynthetic pathways of polysaccharides is involved in the biosynthetic pathways for the nucleotide sugar precursors, the assembly of the repeating monosaccharide units, and the process of polymerization (Gastebois et al., 2009). Therefore, based on the biosynthesis process of polysaccharides and the enrichment results of DEGs GO and KEGG, we selected the related genes in the hypothesized mushroom polysaccharide biosynthetic pathways, β-glucan synthesis in fungi, cell wall stress of the MAPK signaling pathway of yeast, and the glycogen accumulation of the longevity-regulating pathway of yeast to study.

#### Genes Involved in Hypothesized Mushroom Nucleotide Sugar Precursor Biosynthetic Pathways

Previous reports have proposed a mushroom nucleotide sugar precursor biosynthetic pathway based on previous publications (Wang et al., 2017b). This pathway included glycolysis and the synthesis of the nucleoside sugar precursors. According to the results of monosaccharide composition, we speculated that the synthesis of these four polysaccharide precursors includes the synthesis of GDP-Man, GDP-Fuc, UDP-Gal, and UDP-Glu. Therefore, we searched the enzymes involved in these pathways and compared the expression levels of genes encoding these enzymes in different developmental stages of *L. edodes*. The genes encoding these enzymes were expressed in *L. edodes*, which demonstrated that these metabolic pathways exist in *L. edodes*. These enzymes UGP, PGM, and PGI in *G. lucidum* have been proved to be related to the synthesis of submerged mycelial polysaccharides (Wang et al., 2017b). *G. lucidum* and *L. edodes* belong to basidiomycetes. Peng et al. (2015) reported the content percentages of mannose and galactose that were associated with activities of PGI and PGM. The expression levels of the enzymes involved in the synthesis of monosaccharides were not always the same as that of the molar percentage of monosaccharides (Table 2 and Figure 8). For example, the molar percentage of mannose decreased first and then increased at the whole developmental stages and reached the highest in the K3 stage. The related enzymes involved in the synthesis of mannose included GK, PGI, PMI, PMM, and GMP. GK expression levels in K, K1, and K2 stages have been rising and decreased in the K3 stage. The expression levels of PGI and PMI have been rising at the whole developmental stages. PMM expression level in the K1 stage decreased, increased in the K2 stage, and decreased again in the K3 stage. GMP expression level in the K1 stage increased, decreased in the K2 stage, and increased in the K3 stage. This suggested that these enzymes might be coordinated to control the synthesis of monosaccharides.

#### Genes Involved in β-Glucan Synthesis in Fungi

Considering that about 80% of the glucose ratio in the monosaccharide composition and β-configuration glycosidic bonds in four polysaccharide fractions were obtained from development stages K, K1, K2, and K3, we did an analysis of the β-glucan biosynthetic process in fungi to explore the regulatory pathway of glucan production. Then, 143 homologous genes in *L. edodes* were obtained using the Blastp (1e−5) of the sequences in the fungi β-glucan biosynthetic process (GO: 0051274).

D-Glucans from edible mushrooms present diversified chemical structures. The most common type consists of a backbone of β-D-glucose (1→3)-linked frequently and branched at O-6 by β-D-glucose residues as side chains (Ruthes et al., 2015). β-1,3, and β-1,6 glucan synthesis key enzymes have been studied in other fungi. Synthesis of β-1,3-glucan occurs at the plasma membrane. Glucan synthase (GLS) plays a key role in the synthesis of β-1,3-glucan. GLS is thought to contain a catalytic subunit encoded by the two homologous genes FKS1 and FKS2 (Mazur et al., 1995), a regulatory subunit, and the small GTPase Rho1p (Mazur and Baginsky, 1996). The glucan synthesis regulatory protein (SMI1/KNR4) is required for normal levels of β-1,3-glucan. The smi1Δ mutant showed both decreased glucan synthase activity and cell wall β-1,3-glucan content (Guillaume et al., 2004). Gas1p, a GPI-anchored protein localized to the extracellular face of the plasma membrane, has β-1,3-glucanosyltransferase activity and is involved in remodeling (Isabelle et al., 2000). Five expressed unigenes encoding β-1,3-glucan synthesis key enzymes (GLS, GTPase Rho1p, SMI1/KNR4, and Gas1p) were identified in *L. edodes*. GTPase Rho1p gene (*LEGene 05769*) was significantly downregulated in K2 and K3 compared to K1. The downregulation of this gene in the later stage might hinder the synthesis of β-glucan, resulting in the decrease of β-glucan production. Moreover, KRE6 and SKN1 are two key genes in the synthesis of β-1,6-glucan (Han Q. et al., 2019). Five homologous genes of SKN1 and KRE6 in *L. edodes* were identified, which may participate in the synthesis of β-1,6-glucan in *L. edodes*. *LE01Gene04938* was significantly upregulated in K3 stages compared to other stages, which may lead to the increase in β-1,6-glucan in the later stage.

An extensive study about expression of DEGs involved in β-glucan synthesis in fungi has been carried out. Many DEGs were annotated as a negative regulator of sexual conjugation and meiosis, serine/threonine protein kinase, Rho GTPase-activating protein 22 (BEM2), and the heat shock protein. Then, 22 upregulated genes were enriched in two metabolic pathways: galactose metabolism and the longevity-regulating pathway in KvsK2. We searched for the BEM2 protein and found that it was involved in cell wall stress of the MAPK signaling pathway of yeast (Supplementary Figure 1), which affected the activity of FKS and further affected the synthesis of β-glucan and the remodeling of the cell wall. The downregulation of BEM2 expression in the K3 stage may lead to a decrease in β-glucan production. Rim15 was an important gene that participates in the glycogen accumulation of the longevity-regulating pathway of yeast (Supplementary Figure 2). Rim15 was found to integrate signals derived from several different nutrient sensory kinases that transmit information on the availability of different nutrients (including glucose) (Swinnen et al., 2006). The upregulation of Rim15 expression in the K2 stage may lead to an increase of β-glucan production. Therefore, we speculated that these two metabolic pathways may affect the synthesis of LEFP30.

#### Genes Involved in Cell Wall Stress of the MAPK Signaling Pathway-Yeast and Glycogen Accumulation of the Longevity Regulating Pathway-Yeast

Fifteen homologous genes involved in cell wall stress of the MAPK signaling pathway of yeast were identified in *L. edodes.* The cell wall integrity and stress response component WSC gene (*LE01Gene13406*) were significantly upregulated, and the Rho GTPase-activating protein gene (*LE01Gene04358*) was significantly downregulated in K3 compared to K, K1, and K2. There was no significant difference in the expression of other genes. This suggested that the Bem2 gene may play an important role in GLS activity. Seven homologous genes involved in glycogen accumulation of the longevity-regulating pathway of yeast were identified in *L. edodes.* In addition to Rim15, there was no significant difference in the expression of other genes throughout development.

### Hypothesized *L. edodes* Polysaccharide Biosynthetic Pathways

**FIGURE 11:**
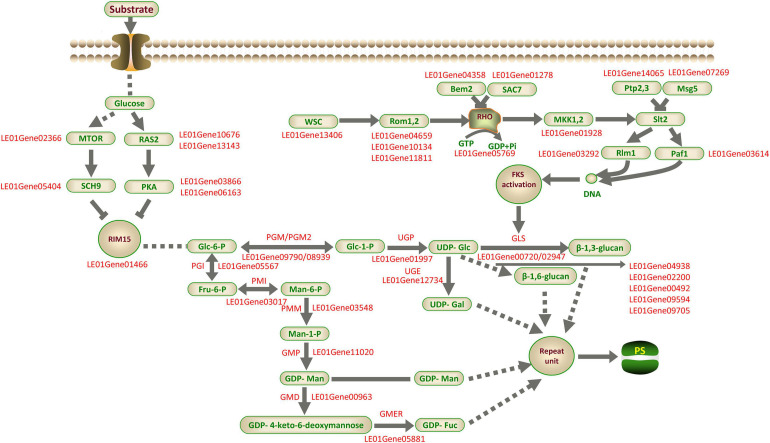
Putative model of LEFP30 biosynthesis in *L. edodes*. The supported and unsupported paths are represented by solid arrows and dashed arrows, respectively. MTOR, Serine/threonine-protein kinase tor2; RAS2, Ras-like protein; SCH9, Serine/threonine-protein kinase SCH9; PKA, cAMP-dependent protein kinase type 1; RIM15, Serine/threonine-protein kinase cek1; PGI, Phosphoglucose isomerase; PMI, Phosphomannose isomerase; PMM, Phosphomannose mutase; GMP, GDP-Man pyrophosphorylase; GMD, GDP-Man dehydratase; GMER, GDP-4-keto-6-deoxymannose epimerase/reductase; PGM/PGM2, Phosphoglucose mutase; UGP, UDP-Glc pyrophosphorylase; UGE, UDP-Gal-4-epimerase; SAC7, Rho-GTPase-activating protein; MAK1,2, MAP kinase kinase; Rlm1, Serum response factor homolog A; Paf1, RNA polymerase II-associated protein; Rom1,2, Rho1 guanine nucleotide exchange factor; RHO, GTP-binding protein; Msg5, Dual specificity protein phosphatase; Ptp2,3, Receptor-type tyrosine-protein phosphatase; Bem2, Beta-chimaerin; GLS,β-1,3-glucan synthase; SMI1/KNR4, Glucan synthesis regulatory protein; Gas1p,β-1,3-glucanosyltransferase; PS, polysaccharide.

Based on the above analysis, we proposed a putative model of LEFP30 biosynthesis in *L. edodes* (Figure 11). This model included the signal transduction pathway of glucose into the cell membrane, the synthesis pathway of the nucleoside precursor, and the regulatory pathway of LEFP30 synthesis.

The study findings can provide theoretical guidance for the metabolic regulation of high-yield LEFP30 in the future. We expect to further verify the conclusion of this study through enzyme activity, metabolomics, and proteomes. This work is expected to advance the research of *L. edodes* polysaccharides to the next level, facilitating the characterization of genes, regulators, and functional markers and also the engineering of the polysaccharide biosynthetic pathway in *L. edodes* or through synthetic biology approaches.

## Data Availability Statement

We uploaded all raw sequencing data on the NCBI Sequence Read Archive (SRA, http://www.ncbi.nlm.nih.gov/Traces/sra). The accession number is PRJNA637998.

## Author Contributions

QT, XS, and YG conceived the study. QL and JC performed the experiments and wrote the manuscript. JL, HY, and LZ analyzed the data. CS, YL, and NJ performed the experiments. All authors contributed to the article and approved the submitted version.

## Conflict of Interest

The authors declare that the research was conducted in the absence of any commercial or financial relationships that could be construed as a potential conflict of interest.

## Footnotes

1 https://github.com/jstjohn/SeqPrep

2 https://github.com/najoshi/sickle

3 http://ccb.jhu.edu/software/hisat2/index.shtml

4 https://www.ncbi.nlm.nih.gov/nuccore/LDAT00000000

5 http://ccb.jhu.edu/software/stringtie/

6 http://deweylab.github.io/RSEM/

7 http://bioconductor.org/packages/stats/bioc/DESeq2/

## Supplementary Material

Primer information were given in Supplementary Table 1. Trimming and read mapping information of the sequences emerged from *L. edodes* at different development stages were given in Supplementary Table 2. Functional annotation of transcriptome data in five databases were given in Supplementary Table 3. GO and KEGG classification information of unigenes were displayed in Supplementary Tables 4, S5, respectively. The detailed information for the identified DEGs of fruiting bodies in different developmental stages was displayed in Supplementary Table 6. GO and KEGG enrichment information of DEGs were displayed in Supplementary Tables 7, 8, respectively. Homologous genes in the fungi β-glucan biosynthetic process (GO: 0051274) were displayed in Supplementary Table 9. The differentially expressed homologous genes involved in β-glucan synthesis in fungi at different developmental stages were shown in Supplementary Table 10. MAPK signaling pathway-yeast was shown in Supplementary Figure 1. Longevity regulating pathway-yeast was shown in Supplementary Figure 2.
