# Supplementary figures and images for: De novo Sequencing and Comparative Transcriptome Analyses Provide First Insights Into Polysaccharide Biosynthesis During Fruiting Body Development of Lentinula edodes
Source: Front Microbiol. 2021 Jul 13;12:627099. doi: 10.3389/fmicb.2021.627099 (PMC8313990; doi:10.3389/fmicb.2021.627099)

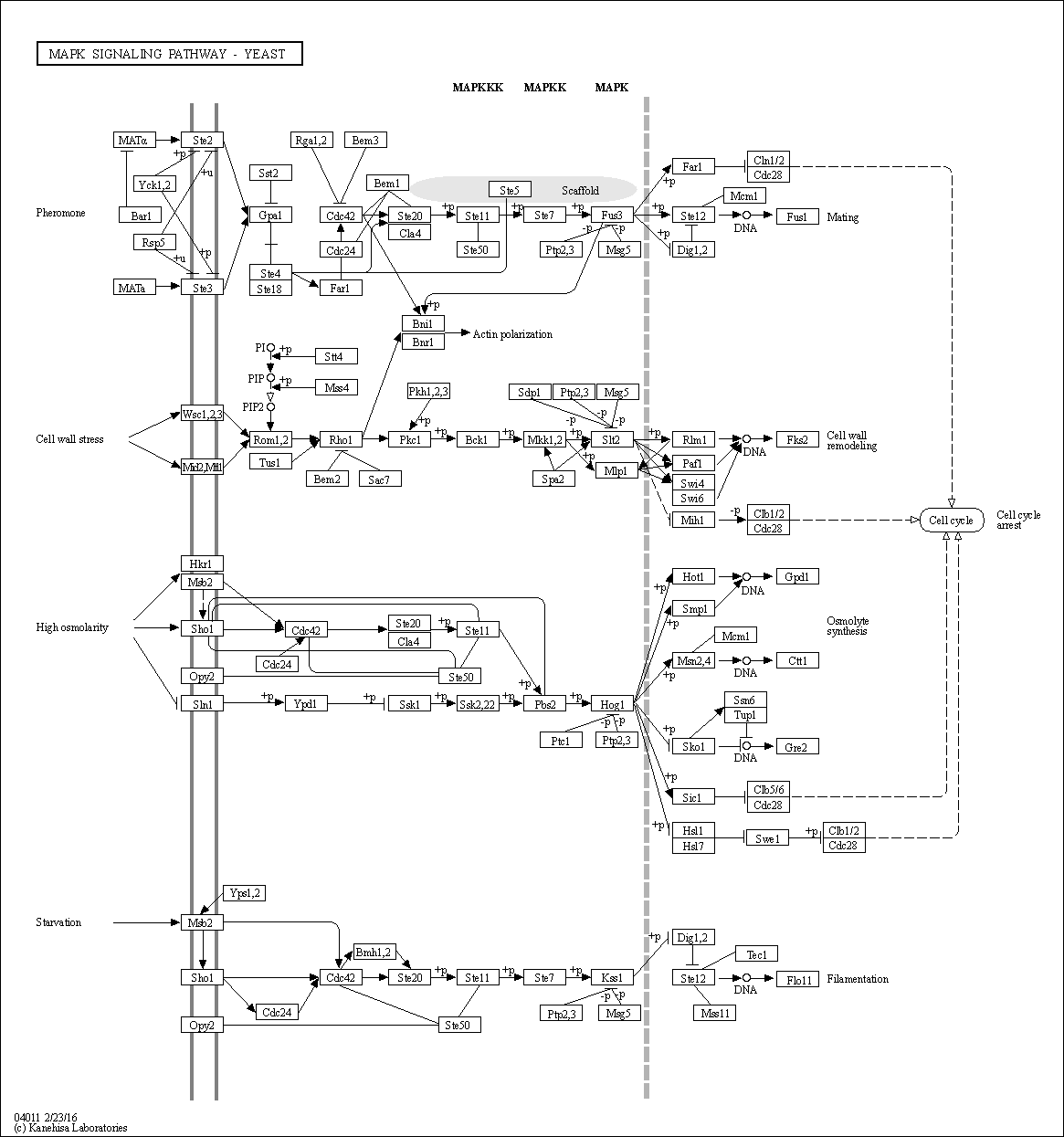

Supplement: Supplementary file 11 [file Image_1.PNG]

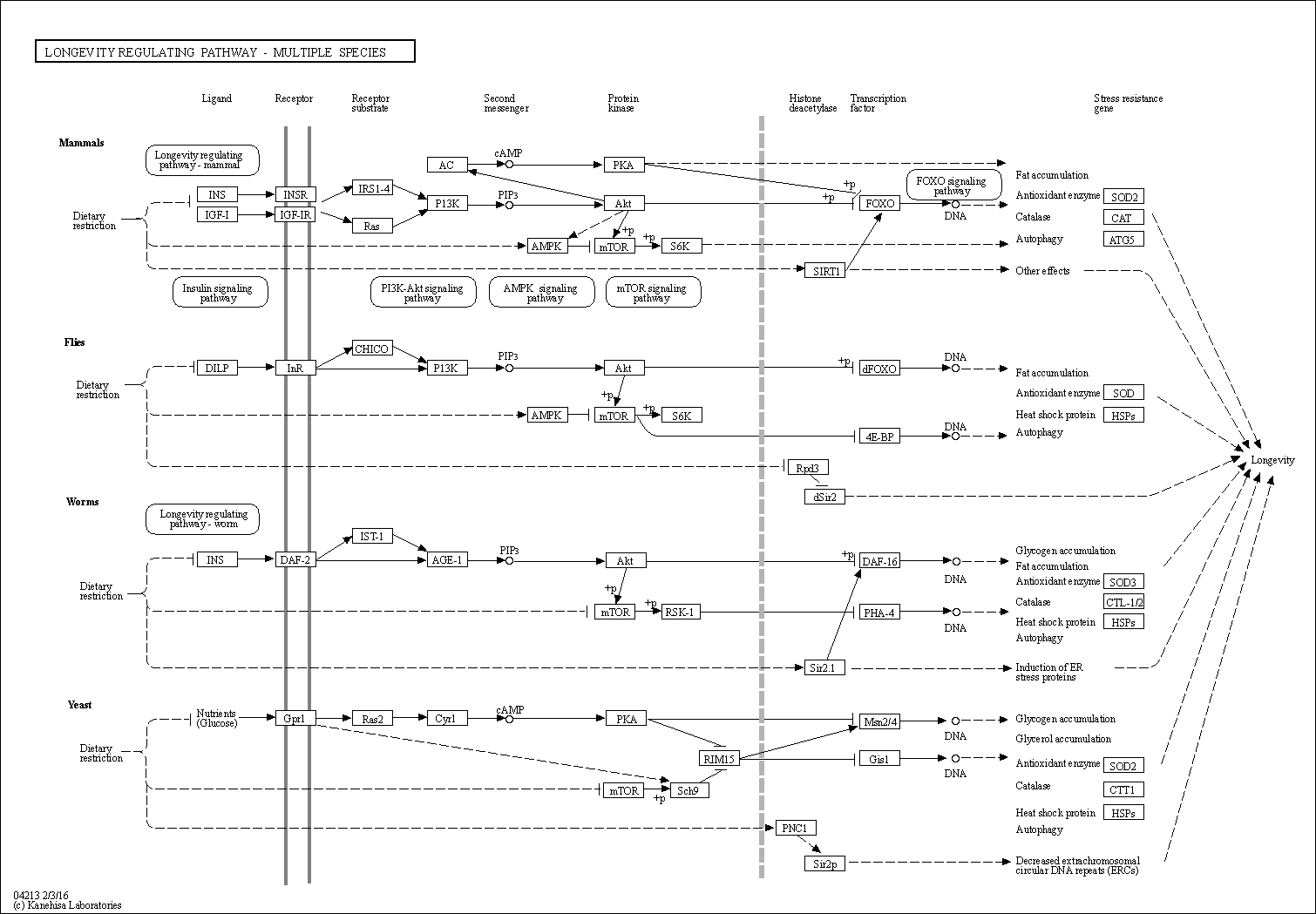

Supplement: Supplementary file 12 [file Image_2.PNG]
